# SynCoPa: Visualizing Connectivity Paths and Synapses Over Detailed Morphologies

**DOI:** 10.3389/fninf.2021.753997

**Published:** 2021-12-27

**Authors:** Sergio E. Galindo, Pablo Toharia, Oscar D. Robles, Luis Pastor

**Affiliations:** ^1^Ciencias de la Computación, Arquitectura de Computado, Lenguajes y Sistemas Informáticos y Estadística e Investigación Operativa, Esc. Tec. Sup. de Ingeniería Informática, Rey Juan Carlos University, Madrid, Spain; ^2^DATSI, ETSIINF, Universidad Politécnica de Madrid, Madrid, Spain; ^3^Center for Computational Simulation, Universidad Politécnica de Madrid, Madrid, Spain

**Keywords:** scientific and data visualization in neuroscience, joint neuron morphology and connectivity visualization, neuron network connectivity visual analytics, bioinformatics visualization, visual analytics in neuroscience

## Abstract

Brain complexity has traditionally fomented the division of neuroscience into somehow separated compartments; the coexistence of the anatomical, physiological, and connectomics points of view is just a paradigmatic example of this situation. However, there are times when it is important to combine some of these standpoints for getting a global picture, like for fully analyzing the morphological and topological features of a specific neuronal circuit. Within this framework, this article presents SynCoPa, a tool designed for bridging gaps among representations by providing techniques that allow combining detailed morphological neuron representations with the visualization of neuron interconnections at the synapse level. SynCoPa has been conceived for the interactive exploration and analysis of the connectivity elements and paths of simple to medium complexity neuronal circuits at the connectome level. This has been done by providing visual metaphors for synapses and interconnection paths, in combination with the representation of detailed neuron morphologies. SynCoPa could be helpful, for example, for establishing or confirming a hypothesis about the spatial distributions of synapses, or for answering questions about the way neurons establish connections or the relationships between connectivity and morphological features. Last, SynCoPa is easily extendable to include functional data provided, for example, by any of the morphologically-detailed simulators available nowadays, such as Neuron and Arbor, for providing a deep insight into the circuits features prior to simulating it, in particular any analysis where it is important to combine morphology, network topology, and physiology.

## 1. Introduction

The last decades have witnessed a renewed interest in neuroscience, fostered by the launching of a number of multidisciplinary research initiatives of wide base and support (Collins and Prabhakar, 2013; Markram et al., 2015). These initiatives have resulted in continuous growth of the number of researchers and laboratories working on different aspects of neuroscience. Furthermore, they have led to an uninterrupted acceleration of the pace at which new findings are being produced. But in spite of the fact that the last decades have seen remarkable advances, there is still a very long way to go before the scientific community can claim to be able to fully understand, explain or replicate the complex mechanisms underlying brain function.

There are many factors behind this situation, which are consequences of the enormous complexity of the nervous system. In order to simplify the problem, researchers have traditionally focused on narrower areas of interest along three main lines: constraining the domain of the variables under study (and therefore, restricting studies to separate fields such as anatomical, structural, functional, etc.); limiting the level of abstraction within certain ranges (like to behavioral, ultrastructural, molecular, etc.), or concentrating on specific brain areas.

Limiting the scope of a particular study is an effective and even mandatory approach when complexity is a major issue. However, its main drawback is that it provides just a partial view, preventing researchers from developing a cohesive understanding of the whole system. For example, well-studied neuronal circuits are reasonably understood today and have led to valid functional models. But both theorists and experimental neuroscientists agree on the need to improve hypotheses about many fundamental aspects, like how the brain store and retrieve information, which often requires a better understanding of the components of the brain, the way these components communicate with each other, and how to relate stimuli with specific behaviors (Evanko and Pastrana, 2013). Also, a deeper understanding of synaptic connectivity (that is, *connectomics*) will certainly help for that kind of goal (Morgan and Lichtman, 2013), although the decoding of the connectome is one of the greatest challenges nowadays. For this purpose, it is necessary to count on tools that allow combining partial results into a unified framework. The availability of powerful and affordable computing systems offers nowadays the possibility of designing and building these tools, something that was out of reach until not that long ago.

There are many areas and examples in brain research that can be cited to illustrate this situation, such as the case of computational neuroscience. For example, simulators are useful tools for many issues, such as posing and validating hypotheses (Bruckner et al., 2009; Lin et al., 2011; Beyer et al., 2013; Garcia-Cantero et al., 2017; Peyser et al., 2017; Galindo et al., 2020). Again, the complexity of the performed simulations has been traditionally limited, following the approaches described above, because of practical, technological, and economic reasons: large scale, high detail, and brain-scale simulations are still out of reach. Consequently, integrative tools are still the best option for getting closer to holistic views.

To address these issues, we created SynCoPa, a tool designed to facilitate the performance of global analysis operations combining data from different domains. SynCoPa nowadays is able to present users with detailed morphological and structural information of neuronal circuits at the connectome level. In addition, SynCoPa is easily extendable to include functional data provided, for example, by any of the morphologically-detailed simulators available nowadays, such as Carnevale and Hines (2006) or Akar et al. (2019). SynCoPa can thus facilitate performing multimodal analysis of medium complexity circuits taking into consideration morphological, topological, and functional data.

The rest of this article is organized as follows: section 2 presents the techniques that have been implemented in SynCoPa, as well as the application itself; section 3 presents practical examples of the analysis tasks that can be accomplished using SynCoPa as well as the solutions we propose; finally, section 4 presents the conclusions and proposals for future work.

### 1.1. Related Work

The study of connectivity was quite important even when the field of neuroimaging was not yet mature (Friston, 1994). The consolidation of this area, as a consequence of the availability of new technologies, led later on to a remarkable increase in the number of publications on functional connectivity, making it a very interesting and challenging topic (Friston, 2011).

Some authors, such as van Dixhoorn et al. (2010), have pointed out how the problem of visualization of functional connectivity lies at the confluence of scientific and information visualization. They consequentially adapted techniques from visual analytics, such as multiple coupled anatomical and abstract views to aid with the iterative exploratory selection of relevant aspects from full data sets.

In this sensex, Ma and Muelder (2013) explain how network analysts are turning to visualization, not just taken as the passive process of producing images from numbers, but as a discipline that creates highly interactive methods that combine visual representations with network analytics to greatly enhance the ability to understand and characterize the networks under consideration. These new methods must address all aspects of network representation, from the fundamental problem of laying out a large graph (that is, how to efficiently provide a node placement or layout that will yield a meaningful graph visualization) to graph analytics and simplification for dynamic graphs. They conclude that the convergence of analysis tools, such as interactive visualization, among others, will lead to powerful visual analytics solutions.

Then, throughout the state of the art, several works focused on visualizing neural connectivity can be found, using different representation techniques and visualization approaches.

One of the most used techniques is based on connectivity matrices, and several works can be cited. The first one is the work of Rubinov and Sporns (2010), who present a Matlab toolbox, which is able to generate a connectivity matrix where its cells have binary information denoting the presence or absence of connections, or even weighted information that represents magnitudes of correlational or causal interactions. These matrices can also deal with directionality in links, although the authors point out that the neuroimaging methods they have access to are unable to directly detect anatomical or causal directionality.

Another matrix-based work to cite is Nordlie and Plesser (2010), who present Connectivity Pattern Tables (CPTs), a 2D compact and schematic representation that tries to show both the strength of connections as well as their spatial structure. The main highlights of CPTs are a clutter-free depiction of connectivity; the ability to represent connectivity at several levels of aggregation, and the CPT's rich information content regarding the spatial structure of connectivity.

Mijalkov et al. (2017) studied the brain connectivity applying techniques from graph analysis theory as a way of integrating visualization mainly based on connectivity matrices. It has to be noticed that the visualizations they build with this Matlab tool do not provide any morphology information.

Another tool based on connectivity matrices is Brainography (LoCastro et al., 2014). It is a tool written in MATLAB that displays the brain and its connectivity, providing many choices for customizing the presentation of results and generating renderings for analysis or publication. This method can display many parts of an atlas in explicitly chosen colors. Using an underlying connectivity matrix, the user can determine whether to include nodes and pipes in the final plot.

Some other works, such as *eConnectome* and *Braincove*, deal with functional information associated with the connectivity data. On one hand, *eConnectome*, developed by He et al. (2011), is a Matlab toolbox intended for mapping and imaging functional connectivity at both the scalp and cortical levels from electroencephalograms and elecotrocorticograms. It provides a platform for imaging brain functional connectivity data and for visualizing functional connectivity patterns over a geometrically realistic scalp or cortical surface. The toolbox shows where, when, and how neuronal assemblies are activated and coordinated. In addition, it also offers users access to integrated connectivity results. Regarding connectivity analysis, they state the importance of estimating the true connectivity pattern among brain regions of interest. Unfortunately, they cannot ensure a correct statistical estimation of the functional connectivity paths between cortical areas. On the other hand, *Braincove*, created by van Dixhoorn et al. (2012), is focused on presenting different coupled visualizations to perform the visual analysis of voxel-wise fMRI connectivity. Large brain networks can be visualized in their anatomical context, and it can be said that it also uses an interactive matrix representation.

Dealing also with functional information, it is worth citing the interesting alternative suggested by Böttger et al. (2014), who developed an edge bundling method with the aim of depicting clear high-resolution pictures of functional brain connectivity data across functional networks in their native anatomical space. It can be said that they build a connectivity matrix as an intermediate representation. It is also relevant to point out that the authors also stressed the importance of the correspondence of the functional connectivity information with the anatomical space.

Some other authors have approached the world of visual analytics, although from very different perspectives. In this way, the work of Fujiwara et al. (2017) present here a visual analytics system designed to enable neuroscientists to compare networks. Their system provides visual tools for comparison at both individual and population levels. The main visualization techniques they use are based on representations of connectivity and node-linkage matrices (both 2D and 3D). On other hand, Euán et al. (2019) created HCC-Vis, an R-Shiny application to explore the results obtained from the development of a hierarchical cluster coherence (HCC). This application is focused on the analysis of connectivity in neuronal networks.

Another work related to visual analytics is the one from Conte et al. (2016), who created a web-based 3D tool with the aim of allowing the interactive exploration of the *intrinsic geometry* of the connectome. This intrinsic geometry is the topological space defined using derived connectomic metrics rather than anatomical features. It represents the brain connectome after the application of non-linear multidimensional data reduction techniques. The resulting node positions do not correspond to their anatomical location, being a measure of the strength of the interaction that each region has with the other ones. This way, the user can explore the entire connectivity network in an uncluttered way, in contrast with a typical highly connected node-link diagram.

It is shown that Mullen et al. (2013) also used 3D in their work. They focused on showing reconstructed source networks over a 3D brain model, with its cortical regions colored according to the labels assigned by an automated atlas labeling procedure. Their visualizations use BrainMovie3D, which generates sequences of images showing event-related information of localized electroencephalogram waveforms.

Finally, certain authors have provided tools to carry on visual interactive analysis. Al-Awami et al. (2014) created Neurolines, a multi-scale abstract visualization technique for the interactive analysis of neurites and their interconnections. Each neurite is represented as a tree structure, based on its real anatomy (and its branching pattern). One of the most interesting capabilities of Neurolines is the possibility of highlighting a selected synapse; in that case, all other synapses between the same two neurites will be displayed as visual links for contextual information. This allows users to quickly see how many synapses are sharing both neurites as well as to follow synaptic chains throughout the dataset.

Another interactive web-technology visualization tool is VIOLA (Senk et al., 2018), which provide different views for the analysis of the activity of spiking neuronal network simulations. It shows individual 2D plots for different spike-count rates with a spatial representation of network activity at the neuron level. The authors also extended it to a 3D view of spatio-temporal activity, also at the neuron level. Although in their work they consider and model different synapse events, they distribute evenly synaptic currents along the entire length of the dendrite shaft or on the spherical-shaped soma, not showing synapses in any of the views. Also, Combrisson et al. (2019) present Visbrain, an open-source software package that provides a set of visualization tools for brain electrophysiology and neuroimaging data analysis. The visualizations it provides range from electrode localization to project data onto the cortical mesh, to connectivity among regions of interest. This connectivity is represented by drawing lines linking nodes (which may be EEG sensors, not necessarily neurons).

The tool presented in this article allows to analyzing neural connectivity in combination with detailed morphologies. To the authors' knowledge, no other previous work presents any tool able to accomplish the same tasks that SynCoPa performs, making it difficult any direct comparison.

## 2. Materials and Methods

Understanding the brain is a quite challenging task. Usually, in order to cope with its complexity, its study has been carried out by faceting the data and focusing research efforts in one of these facets or domains (such as morphology, physiology, or connectomics). This approach reduces the amount and types of data under analysis, making its processing, analysis and storage easier, and facilitating the acquisition of domain-specific knowledge. On the other hand, bridging the gap among domains and understanding their interrelationships is also essential for global analysis and understanding of the brain. This seemed to be unfeasible a few years ago; however, it has already started to be possible thanks to the remarkable advances in technology produced during the last years and the computing capabilities available nowadays.

This article presents a step in this direction, contributing to bridging this gap by providing techniques and tools that facilitate performing a combined visual analysis of two of these domains: morphology and connectivity (with the possibility of including also physiological information).

More specifically, the work presented in this article focuses on two analysis tasks. The first one is centered on the interactive analysis of the relationships among the detailed morphologies of a neuronal circuit and its synapses' spatial location and attributes. This could help to study the combined effect of the spatial location of synapses and their features in circuit behavior, facilitating establishing or confirming a hypothesis about the existence of patterns or correlations between these issues in specific circuits.

The second analysis task considered here addresses the study of the multiple paths connecting neurons in low to medium complexity circuits and their most relevant features (which includes the morphological features of both the neurites and synapses involved in each path). This analysis can provide a deep knowledge of the connectivity of specific circuits, answering questions about how the connection between different neurons has been established or the relationships among morphological features and multiple synapses within specific circuits.

The approach proposed in this article is based on the exploitation of interactive visual exploration tools for the analysis tasks described above, which allows taking advantage of the analysis capabilities that the human visual system provides. Adding fluid interactivity (navigation, selection, filtering, etc.) on top of expressive visualization metaphors permits users to handle larger and more complex data sets, letting them manage how much data or detail is being displayed at any given moment. In this sense, we have followed Shneiderman (1996) mantra of “Overview first, zoom and filter, details on demand.” To the authors' knowledge, there are not any other previous work that provides this type of combined visual analysis of detailed morphologies and connectivity information.

In order to show the feasibility and potential of the proposed approach, all these methods have been implemented in a functional prototype application: SynCoPa. This tool is also used to show how the proposed methods can be applied to the aforementioned analysis tasks and its main functional requirements can be described as follows:

- FR1. The user should be able to load data (morphologies and connectivity)
- FR2. The user should be able to visualize the synapses spatially, tune the visualization parameters (color, opacity, etc.), and apply filtering operations to perceive meaningful patterns.
- FR3. The user should be able to visualize synapses (pre and post positions if available on the data) on top of morphologies and adjust visualization parameters to enhance interpretation.
- FR4. The user should be able to select two sets of neurons and highlight the paths connecting them.

The rest of this section is organized as follows: section 2.1 describes the type of data used in this work; section 2.2 describes the morphological visualization used; section 2.3 focuses on the particle-based rendering technique used for the different implementations; 2.4 and 2.5 explain the design decisions taken for both analysis tasks posed before (synapses and connectivity paths), and last, section 2.6 shows how all these techniques have been implemented in the SynCoPa application.

### 2.1. Data Domain

In this section, the type of data used in this work is presented and described. As the proposed approach aims to help with tasks combining morphological and connectivity information, both types of data have to be considered. Each of them has its own specific features and problems, but for joint analysis, it becomes mandatory that both types of data (morphological and *connectomical*) are coherent and there is a well-established relationship between them.

**Figure 1:**
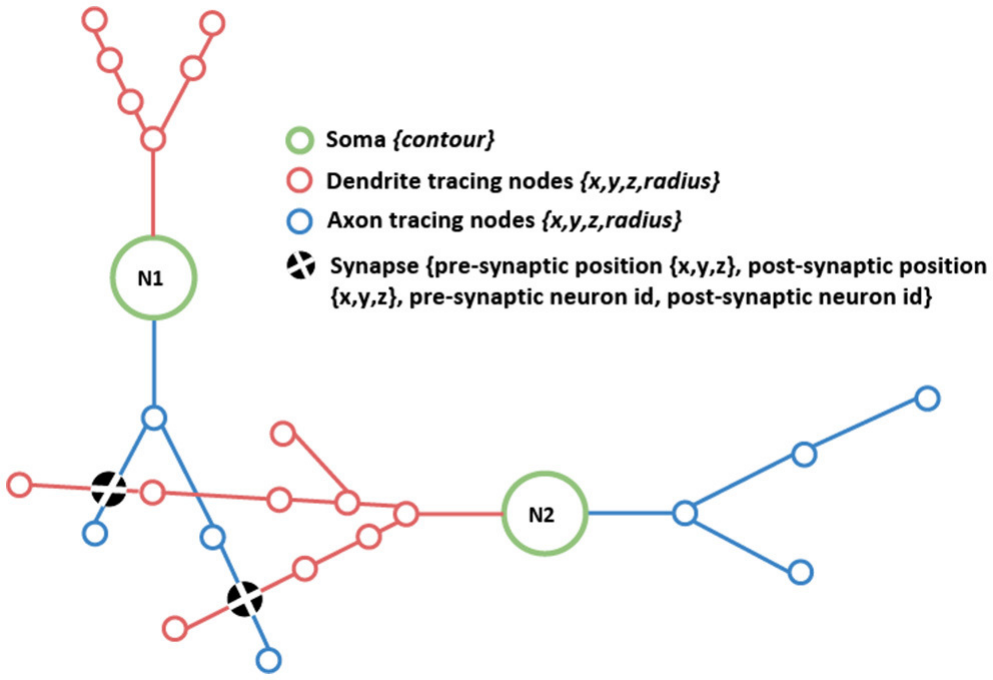
Schematic drawing depicting the morphological data components addressed in this work: detailed neuronal morphologies connected through their shared synapses. The figure shows the different components, such as the neuronal somas (green circles); axonal and dendritic tracings (depicted in blue and red, respectively), and synapses, represented as black filled circles indicating the location of the connection. Axonal and dendritic tracings are defined through sets of tracing nodes.

On one hand, the morphological data has to provide a detailed representation of the neurons' structure. This information could be obtained from images captured using different microscopy technologies, and once the images are captured, different manual, automatic, or semi-automatically tracing algorithms can be used. For each neuron, the output of this process can either be a complex polygonal mesh or a simpler cell skeleton, being the latter the most common one. This skeleton is composed of tracing nodes and the connections between them, providing a tree-like representation, where the spatial location of each node is kept (see Figure 1). Additionally, the radius of the dendrites and axons can be measured at each tracing node and kept with them, in order to have a more accurate representation of the cells. Alternatively, neuron skeletons can be synthesized, even departing from real (or plausible) data.

On the other hand, the connectivity data has to provide information about the existing synapses, their location in space, and the connecting neurons involved, depicted in Figure 1 with black filled circles. This information is crucial to enable a combined analysis of the connectivity paths and the detailed morphologies.

Some data sets represent synapses using pre-synaptic and post-synaptic information. In this work, we have used this type of data, as it is more detailed, although the methods could be simplified to work with just one general synaptic position.

In addition, other synapsis features can also be taken into account and added to the analysis. For instance, in this work, we use the synapse type (axo-dendritic, axo-somatic, dendro-dendritic, etc), but the proposed methods could also be easily applied to other variables, both categorical or numerical.

### 2.2. Morphological Visualization

As stated before, this article proposes a visual analysis method for neuronal circuits that combines morphological data and connectivity information. In order to achieve this goal, it is necessary to be able to render first the cell morphologies. As explained in the previous section, these neurons' morphologies are usually modeled as skeletons composed of hierarchical polylines (connected tracing points). These skeletons start at the soma, which will be the root node of a tree-like structure, see Figure 1. This data scheme matches the SWC file format, a *de facto* standard for storing neuron morphologies in neuroscience (Cannon et al., 1998).

In order to render these skeletons, our method uses NeuroLOTs (Garcia-Cantero et al., 2017), an open source software library with an open API that provides the necessary components for the realistic rendering of detailed morphologies. This software library generates on-the-fly meshes with a high level of detail. Other morphology renderers, such as RTNeuron (Hernando et al., 2013), or using simple plain cylinder-based visualization, among other options, could have been applied here. However, NeuroLOTs has been the selected solution due to its convenient features, such as the adaptive level of detail it provides, the simplicity of use, and our previous knowledge of its API. In any case, the proposed approach could be successfully applied using any other rendering software.

### 2.3. Particle Rendering

In this work, we propose representing the synapse positions and connecting paths using a computer graphics technique called particle-based rendering. This method is based on displaying a large number of computationally cheap elements that are treated independently, but which can be combined and integrated for creating complex visual effects (Hastings et al., 2009).

Using particle-based rendering allows fine control of every displayed element, making it possible to use the particles' visual properties for encoding the main attributes of the elements being represented. In this case, the selected visual properties are related to color and brightness attributes (hue, brightness, saturation, and transparency), being useful for encoding both categorical and quantitative attributes. The application of this type of encoding to synapse visualization is presented in section 2.4.

Particles can be successfully used for representing larger scale environments by rendering them a such a way that they are perceived as being part of the same structure (taking advantage of the laws of proximity and similarity from the Gestalt principles; Koffka, 2014). Here, we take advantage of this property when representing a large number of synapses (section 2.4) or connectivity paths (section 2.5.2). In addition, particles are also suitable for creating complex animation effects, as they are very efficient from the computational point of view. For instance, this allows creating the perception of a “flow” along a specific path. In this work, we propose to use this effect for representing connectivity paths in a dynamic way, enhancing the perception of the paths and their direction (see section 2.5.3).

To achieve all these goals, particle rendering has been implemented here using an improved version of PReFr (Galindo et al., 2015), a general-purpose framework that provides a high level abstraction API for controlling how particles behave and appear. PReFr has been successfully applied to the purpose of visualizing neuronal circuits' simulations (Galindo et al., 2016). Having the capability of rendering up to one million particles on multi-core CPUs, PReFr serves as a stable base for the implementation of the methods proposed in this article.

In this work, we combine the visualization of the neurons' morphologies with the visualization of connectivity (synapses and paths). For the former, we use mesh-based rendering (detailed in section 2.2) while for the latter, we propose using particle-based rendering. The combination of these two types of rendering techniques can lead to visual artifacts when particles intersect with the triangles of the rendered morphologies. In order to alleviate this problem, in SynCoPa we have implemented “soft particles,” a technique for softening particles' edges when crossing geometry (Lorach, 2007).

Another issue that arises when using particles is how to combine them to achieve the desired effect. This typically means deciding which colors and transparency will be used. Both of these parameters interfere with one another, which means that they are not completely separable and have to be chosen carefully. Moreover, dealing with transparency (alpha blending) is highly relevant in particle-based rendering, as it can have a huge impact on the perception of different particles as groups or as a whole. Through different operations, alpha blending allows mixing colors of a large number of semi-transparent elements. This composition of hundreds or even thousands of elements on the screen with alpha blending produces an effect of visual aggregation. As a result, this effect can be used for understanding the concentration of elements and for detecting spatial patterns in complex data, as proposed in section 2.4.

Specifically, regarding alpha blending, there are several ways of composing the final color of a pixel. In this work, we propose using two of the most typical methods: “traditional,” or regular alpha blending (TAB), which composes the image by weighting the color results from the previous layers; and “accumulative,” or additive alpha blending (AAB), which adds the color results until they visually saturate colors to white. On one hand, TAB preserves color coherence for data representations and will enhance the perception of depth. On the other hand, AAB can be used to summarize the concentration of elements, as the color variations will be brighter and with more saturated colors.

### 2.4. Synaptic Visualization

An average neuron has thousands of synapses (Sporns et al., 2005). The representation and interactive visualization of detailed morphologies and connectome details of non-trivial neuronal circuits is therefore a challenge, both from the computational and the visualization points of view. Computationally, it is necessary to display a large number of primitives in a short time for guaranteeing smooth interactivity. From the visualization point of view, it is also essential to reduce visual clutter to a minimum for facilitating the operator analysis tasks.

**Figure 2:**
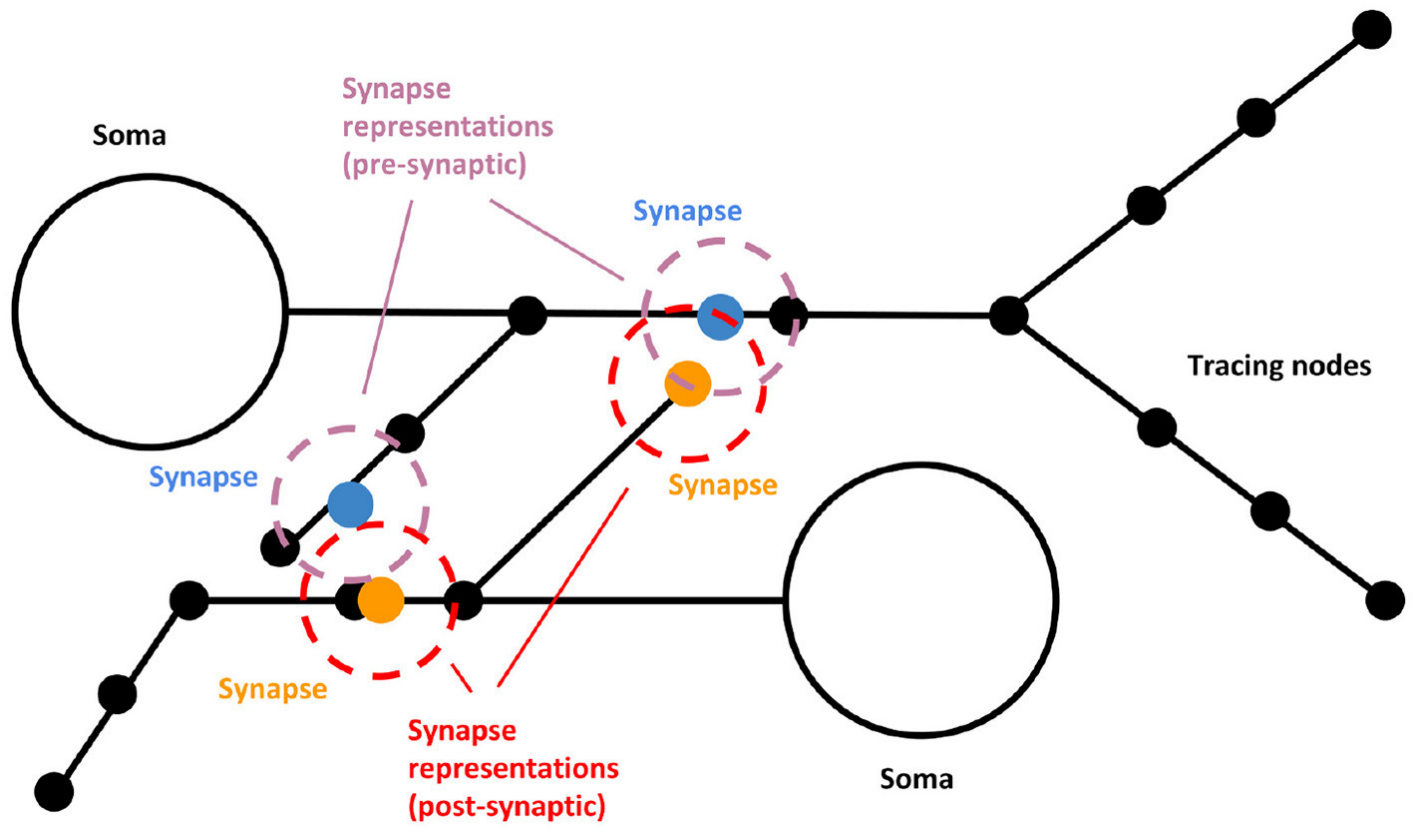
Visual representation scheme for synapses. Two static particles (blue and orange, for the pre- and post-synaptic points) are placed at every synapse position. The representation of the pre and post-synaptic parts (purple and red, respectively) might present differences in color, size, and transparency. When the morphology is rendered, synapses appear surrounding the given axon or dendrite mesh.

As commented before, SynCoPa uses particle rendering for coping with these problems. Following Figure 2, two static particles are placed at every synapse position, one for the pre-synaptic position and another for the post-synaptic one. They are encoded using different colors, so they can be easily distinguished. The user can adjust the size of the particles. This size will vary accordingly with the camera position, as the final 2D image is rendered using a typical rasterization approach based on a perspective projection. In the case of synapses, it can be tuned to create, produce different visual aggregations, allowing to help discover visual patterns and understand their spatial distribution. The use of particle rendering combined with alpha blending and translucency allows decreasing clutter by facilitating visual aggregation. In addition, particle rendering allows using other visual channels (see section 2.3), such as color, size, and transparency, for encoding different synapse attributes. This way, other features, such as conductance, efficiency, synapse type, state, etc., can be mapped to the visual representation of the synapse. Specifically, SynCoPa provides a way of mapping a chosen property (quantitative or qualitative) to a color palette that can be user-defined, producing a variety of colors ranging from the lowest value to the highest one in the palette, and making it easier to visually detect the existence of spatial patterns.

Finally, SynCoPa also depicts a histogram that helps the user to understand the distribution of the attributes' values according to the selected color mapping.

### 2.5. Connectivity Paths Visualization

Displaying synapse positions in a neuronal circuit is just a first step, not being particularly useful when the number of synapses grows beyond an easily reachable limit. However, SynCoPa has other resources that can help with the analysis of how neurons interconnect, highlighting connectivity paths so that they can be clearly distinguished. They will be described in this section.

Let a neurite section be the set of morphological nodes located between two consecutive neurite bifurcations. Then, a connectivity path between two neurons is formed by the neurite sections involved in the connection of these two neurons, from which one acts as pre-synaptic and the other, as post-synaptic. In order to create and visualize a path, all possible routes from one neuron to another have to be computed using their shared synapses as connecting points. This can be easily extended to the connectivity path between two groups of neurons, where these two groups can potentially overlap.

In order to provide mechanisms for the visual analysis and exploration of connectivity within detailed neuronal morphologies, we have to also take into account neural tracings. How to show a large number of synapses, along with their neurons' detailed morphologies and their connections, is one of the main challenges to tackle, and the remarkable complexity of representing both neuronal morphologies and the way they interconnect aggravates the creation of a visual codification that facilitates grasping easily how a neuron connects to others.

In this sense, graph-based and matrix-based visual representations are typical approaches that can help in the task of analyzing connectivity paths but would not be enough to face the combined visual analysis of connectivity and detailed morphologies that we propose in this article, since they cannot represent the latter. To cover this gap, SynCoPa proposes a method to highlight the morphological elements involved in the connectivity paths between two or more neurons using particle rendering. The main features of the proposed methods are presented below.

#### 2.5.1. Path Generation

A neural circuit *C* can be defined as a set of *M* neurons *N*_*i*_ and a set of zero or more synapses *S*_*ij*_ for each pair of neurons *i* and *j*, where neuron *i* acts as pre-synaptic and neuron *j* as post-synaptic. The number of shared synapses between a pair of neurons can differ widely.

Given two neurons *N*_*PRE*_ and *N*_*POST*_, if the number of synapses they have in common is greater than 0, there should be a direct path *p* composed of neurite sections going from the soma of *N*_*PRE*_ to the soma of *N*_*POST*_:


(1) $$\begin{array}{l} \exists p\left(N_{PRE},N_{POST}\right)\Leftrightarrow |S_{PRE,POST}|>0 \end{array}$$

When the set of synapses involved in the connection has been identified, the existing paths are computed using the intermediate neurites sections, including the ones that are closer to the soma. To do this, the easiest algorithm is to start from each synapse and trace its path back to the soma, going up through the neural morphological tree-like hierarchy until reaching the root node. This works well for both axonal and dendritic sections. All these synapses and sections are stored as the working set (from now on WS) to be used for performing the highlighting of the paths in later steps.

In general, the position of synapses does not match the position of an existing tracing node. In that case, after computing the paths, the segments of the WS that contain synapses are trimmed to remove the nodes that should not belong to the path. This adjustment makes the path representation more faithful to the actual path. In addition, we avoid recomputing and sampling preexistent neuritic section paths for all the involved neuron morphologies in order to reduce the complexity of the processes.

#### 2.5.2. Static Visualization

When the potential paths connecting neurons have been identified, their neurite sections are classified, so that the ones involved in the connection between neurons can be highlighted from the others. SynCoPa uses particle rendering for the creation of a halo effect surrounding the involved neurite sections by placing particles all along the connecting path. For that purpose, particles sizes are set to a default fixed value that fills the neuritic segments. This value can be changed by the user to emphasize or diminish the effect of the path highlighting. Thanks to the transparency composition resulting from the use of alpha blending (discussed in section 2.3), all these particles will benefit from the visual aggregation, which allows the user to have the impression of a continuous filament instead of perceiving individual elements.

In order to achieve this, and considering that neuritic sections are stored as polylines, an homogeneous sampling is carried out to compute the positions of the particles. Linear interpolation is done based on distance, which generates a plausible visual effect with a low CPU cost. The sampling distance between particles is equal to their radius, which makes them located at the same Euclidean distance throughout the neurites' polyline. Depending on the angle of the bifurcations, it is possible to find particles placed closer to one another due to the shorter euclidean distance through the polyline. Nevertheless, it does not alter the highlighting effect while preserving a low sampling computational cost.

Unlike many other techniques that could be used to perform this highlighting effect, such as shading effects or image post-processing, the use of particle rendering allows decoupling this step from the represented geometry, simplifying the rendering process. This decoupling would allow using of any other neuronal geometry renderer.

**Figure 3:**
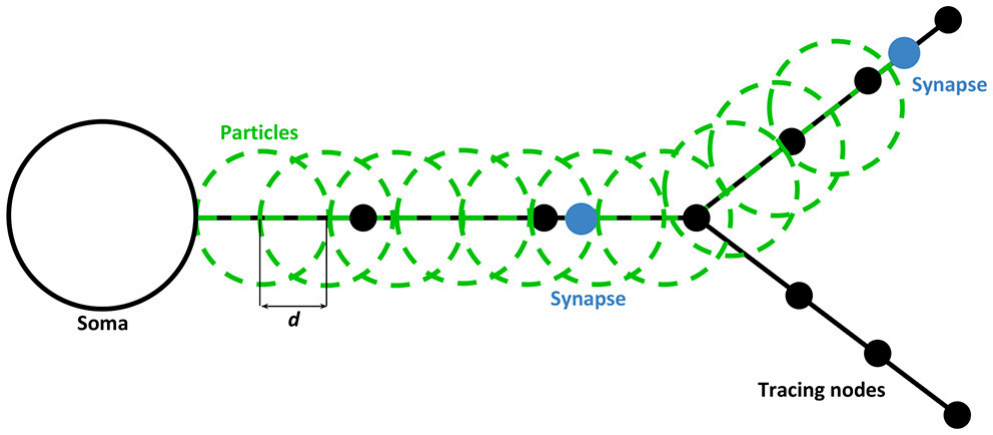
Static path-visualization scheme. Particles (in green) are placed along the neurite sections of the WS from the root node (closest to soma) to the last node just before the last synapse position of each branch. All particles are separated by a distance of *d*, creating the effect of continuity across the highlighted paths. By default, this distance is the radius of the particle, assuring the overlapping of circles defined in the geometry. This, along with the alpha blending, creates the perception of a homogeneous path. The process for both pre and post-synaptic paths is the same.

Figure 3 represents a piece of a neuron with its tracing nodes (black circles) and an example of how particles are positioned along the neuritic segments involved in the connection, which are represented with a blue circle. As it is represented in the figure, the coordinates of the particles are computed using the polylines connecting the nodes and applying a displacement between each particle of a fixed distance (*d*), which depends on the size chosen for the particles. In this sense, particles positions and movements (for dynamic visualization in the next Section) are always interpolated using the paths, that were previously built using the morphological nodes of the neurons' neurites. This ensures that particles will always be located or move on top of and along the neurites.

#### 2.5.3. Dynamic Visualization

The effectiveness of the static visualization previously proposed can be compromised in complex scenarios with a high density of morphologies, so that occlusions and visual clutter might hinder the understanding of the scene. As a result, it becomes necessary to provide mechanisms to emphasize the highlighting of the connectivity paths even on high density scenarios.

As the main visual channels are already being used to represent and identify the paths, a more powerful characterization of them must be used in order to ease their visual analysis and avoid mismatches. The use of motion fosters a “pop-out” effect, that can help to understand the scene as well as to differentiate elements (Bartram, 1997). Through the use of the motion visual channel, the perception of the connecting branches can be reinforced with an animation that stands out the paths at a glance.

SynCoPa uses particles to create the effect of a moving impulse between connected neurons. A set of particle emitters place particles with a short lifetime along a connectivity path, creating this effect of an impulse that moves along the connected sections from soma to soma. This technique enhances the highlighting of the paths and makes it easier to understand the connectivity direction.

Since neuritic sections are stored as polylines, the emitters move throughout them taking into account the linear interpolation of the distance along each polyline. Hence, the position of each mobile emitter is computed using its previous position and a given velocity value. Particles are activated after the displacement of the emitter has been resolved, using the new position as the source of emission to produce an animation along with the subsequent frames.

In order to boost the perception of these moving impulses, a trail effect has been added improving also the comprehension of the connection directionality. This way, the trail effect enhances the path highlighting mechanism, with better differentiation of the highlighted paths from the rest ones. It can be said that the trail effect is also useful to strengthen the perception of connection directionality in static images, as it represents previous positions.

**Figure 4:**
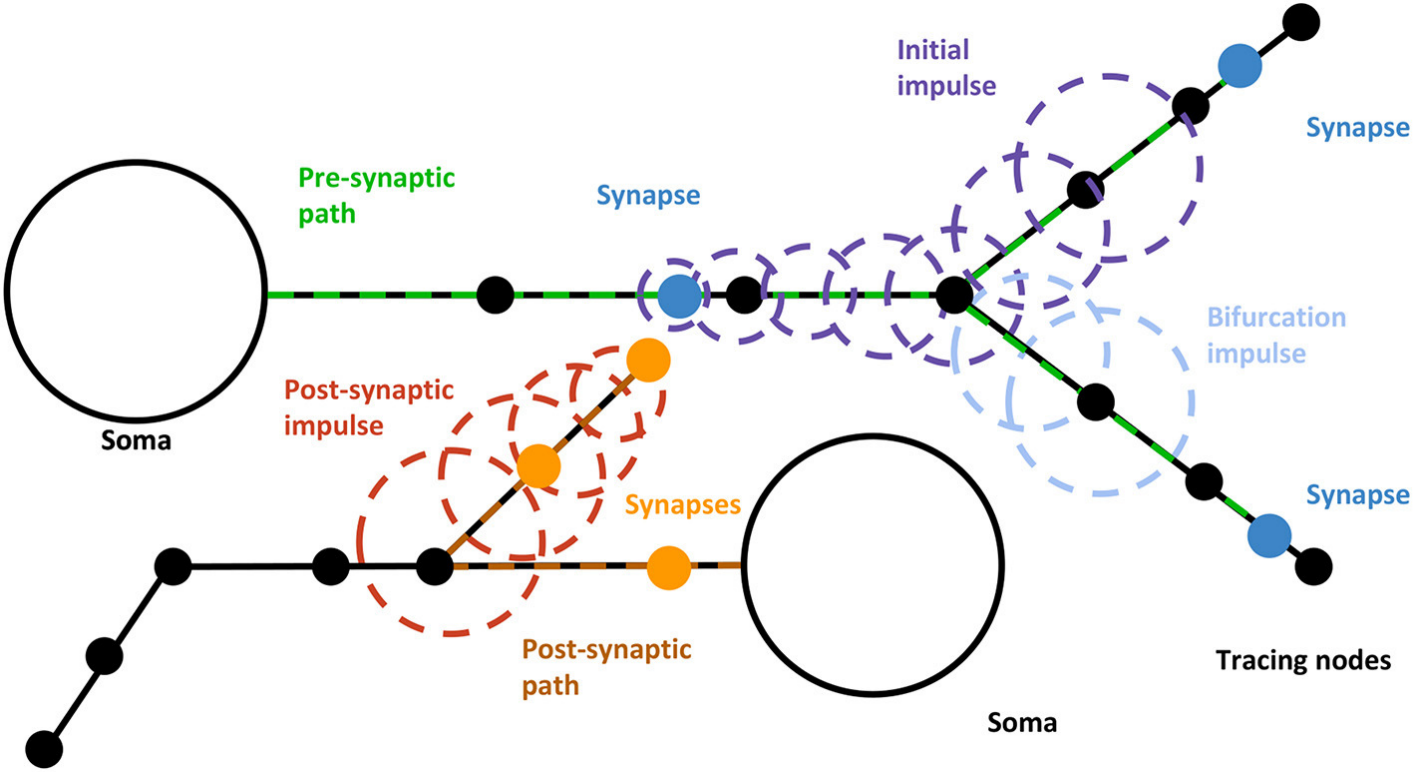
Dynamic paths visualization procedure. The initial impulse moves from the neuron's soma following a certain path along the dendrites, producing other impulses whenever a synapse or bifurcation is reached. When a bifurcation with both branches included in WS is reached, a pre-synaptic impulse (yellow) is created from the bifurcation node to the end of the current branch. When a synapse is reached, a new impulse (light blue) is generated following the path from the synapse position to the post-synaptic neuron's soma. Pre-Synaptic impulses can generate both derived bifurcation and postsynaptic impulses.

Particles are created with a bounded lifetime to harmonize the visual effect created by the decay, remaining at the same positions where they were sampled until they disappear. As the emitter is being moved, particles are homogeneously placed in between the previous and new positions to create the trail effect. This is a way to minimize the impact of either low frame rates or too high velocity, or both things at the same time, because any of these situations could lead to a concentration of sampled particles surrounding the new position instead of producing a uniform sampling. Figure 4 shows how this movement and the sampling process are done.

Once the emitter has been moved, the algorithm checks if any relevant morphological event (i.e., bifurcations, synapses, or path ends) is present along the traveled path. It can be considered that these events happen at a determined time whenever an emitter reaches its position through the polyline. When passing through one of them, the proposed algorithm will perform one of the following actions:

- Bifurcations: a mobile pre-synaptic emitter will be created for each branch contained in WS other than the parent one. Therefore, the activation of the emitter is modeled as a hierarchy. This way, every time an emitter reaches a bifurcation contained in WS, it creates or activates subsequent emitters following its remaining branches.
- Synapses: an emitter will be created so that it will follow a path along the post-synaptic neuron from the synapse to the soma. Following the paths in these directions, there are no bifurcations, so these emitters will not split when reaching them. In order to distinguish these impulses from the pre-synaptic ones, the impulses can be rendered with a different color.
- Path-ends: emitters are deactivated in order to suspend their emission and disappear from view. Path-ends affect both bifurcations and synapse emitters.

Both bifurcation and post-synaptic path emitters are turned off until they are reactivated when the parent emitter passes through the corresponding morphological feature. In case a child emitter's path was longer than its parent's (being therefore not available for reactivation), a new emitter would be allocated in order to maintain the cascade effect.

So, the dynamic highlighting of paths creates a cascade effect from the pre-synaptic soma to the post-synaptic one passing through all the paths computed and taking into account the shared synapses. This effect provides a clear perception of the connectivity directions between related neurons.

### 2.6. SynCoPa Tool

**Figure 5:**
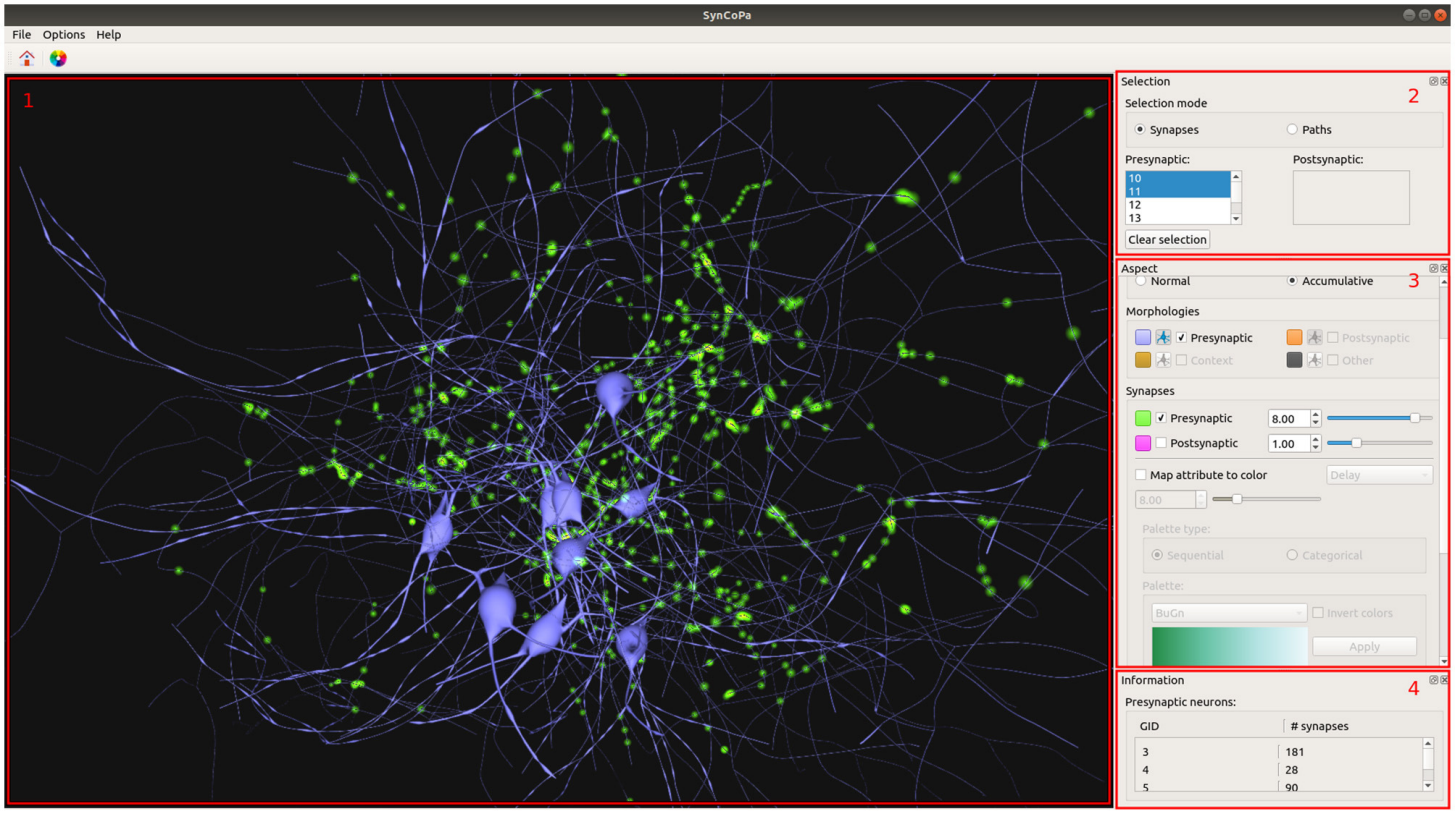
SynCoPa application Graphic User Interface (GUI). 1) 3D Visualizer displaying synapses distribution of several neurons colored in blue. Due to the visual aggregation of particles, the application will show areas with different levels of lightness, where the lighter the representation, the higher the concentration of synapses (in this case, represented in green). 2) Selection Control widget. 3) Aspect configuration widget. 4) Information Summary Widget showing the number of synapses per neuron.

SynCoPa is a desktop application that we have designed to highlight the strengths of the methods explained in the previous sections, with the aim of helping a user to perform a combined visual analysis of morphology and connectivity. To do this, SynCoPa's Graphic User Interface (GUI) is made up of four widgets. Figure 5 shows these four main widgets: 3D Visualizer, Selection Control, Visual Aspect Configuration, and Information Summary.

The first one is the 3D Visualizer (as shown in Figure 5 in the left hand side area); it displays the morphologies, synapses, and paths in a three-dimensional interactive scene. This widget allows to explore the data and interact with it, by means of camera rotation and zooming. This way, the user is able to focus on specific desired areas at any time.

When the set of selected neurons is modified, SynCoPa automatically adjusts the camera to fit in the screen the whole set of synapses. This camera adjustment is done with an animated transition instead of a jump cut so that the user does not lose the overall context.

The second one is the Selection Control widget (shown in Figure 5, upper right). It provides controls to manage the visualization modes and the user's current selection. There are two possible modes, according to the two analysis tasks posed at the beginning of this section 2: synapses and paths, where the synapses mode is used by default. The selected neurons, both pre and post-synaptic, can be defined through two neuron identifiers (GID) lists. When the user selects one or more post-synaptic neurons, the viewer will display the complete morphologies and reduce the paths and synapses to only those shared by the selected neurons. The user will be able to select different neurons acting as pre-synaptic by simply clicking on their GID.

The third one is the Aspect Configuration Widget (that can be seen in Figure 5, middle right); it provides tools to control how morphologies are displayed, as to assign colors to the different elements or to decide to view all the morphologies of the neurons or only the somas. Then, it also contains the controls to tune the color, size, and transparency of the particles used for synapses, as well as the particles used for paths. All these parameters can be set independently making the visualization highly configurable. This widget also includes controls to filter synapses based on their mapped attribute values (see section 2.4).

The user can map an attribute of the synapses to color in order to analyze the spatial distribution of values. In addition, the user can define a range of values for that attribute to focus their attention on a subset by hiding synapses outside that range. When this operation is performed, a histogram representing the selected attribute distribution is depicted on top of the color palette, with a double slider placed below that allows to do the filtering operation that focuses on specific data.

The flexibility of being able to assign different colors to the different elements that are displayed is complemented with the controls placed in the main window to change the background since its color can affect the visual perception of the different elements that are displayed in the 3D Visualizer.

The fourth one is the Information Summary Widget (that appears in Figure 5, bottom right), which provides a summarized view of the data available for the visualized neurons, such as the number of synapses present on the scene. This widget could be easily expanded to display more relevant information about the scene and its elements.

Finally, it can be said that SynCoPa has been built using Qt 5 and it requires to have a GPU with at least OpenGL 4.0 compatibility in order to deploy NeuroLOTs' tessellation (Garcia-Cantero et al., 2017) and specifically its level of detail control. Currently, SynCoPa is available for Linux and MacOS operating systems and binaries can be downloaded from https://vg-lab.es/apps/syncopa/. It is planned to release Windows binaries and to make the code open source in the near future. Regarding the data, SynCoPa currently allows loading data using BlueConfig files, although we are working on providing data loading using other file formats, such as SONATA.

## 3. Results and Discussion

In this Section, we present practical examples of the analysis tasks and the solutions proposed in this article. To do so, we have selected a dataset composed of around 8,000 neurons and 170,407 synapses. This dataset is a subset of a larger one provided by The Blue Brain Project (Markram, 2006) which represents 7 columns of the human cortex.

The results presented in this section are split into four categories and each one describes an example use case. These examples cover the two main analysis tasks this article focuses on, as well as the proposed methods to tackle them. The first use case is based on the visual exploration of the synapses present in a data-set and how they relate to the morphologies (section 3.1). The second scenario details how to visualize paths statically between a pre-synaptic neuron and one or more post-synaptic neurons (section 3.2). The third one describes the synapse and path filtering process based on the synaptic properties for a specific simulation time (section 3.3). The last one describes how the use of dynamic highlighting for path visualization can enhance the static approach and exemplifies how animation can benefit path visualization (section 3.4).

Finally, after presenting these four use case scenarios, section 3.5 is devoted to discussing the scope and limitations of both the method and software application presented in this work.

### 3.1. Synapses

The following use case describes an example of how our method can be used for exploring a synapses' distribution. This visual exploration can be done both with or without the context provided by the neuron morphologies. Counting with neuron morphologies allows posing questions regarding the relationship between the synapses spatial distribution and different morphological features. On the other hand, not counting with neuron morphologies can alleviate visual clutter problems while still being able to detect spatial patterns in synapses positions.

An example of a possible workflow for this use case could be as follows: 1) The user runs the application and loads a circuit, displaying all the available synapses; 2) Then, the user can adjust both the size and opacity of the particle-based representations used for synapses. Fine tuning the opacity will allow the user to reduce the amount of visual aggregation when the number of synapses is large, therefore reducing visual clutter and improving the perception of patterns or clusters; 3) The user can select one or more neurons from the list. This will update the view, which will show then only the synapses belonging to the selected neurons. When the user selects different neurons, SynCoPa will pan and zoom the camera viewpoint to use the maximum screen space available. Instead of using a hard cut, we use a camera animation which will help the user to keep track of the spatial relationship between the previous and current selection; 4) At any time, the user can perform the following operations: modify the visualization parameters, interactively navigate the scene with the appropriate 3D camera controls, and display the neuron morphologies along with the synapses representations.

**Figure 6:**
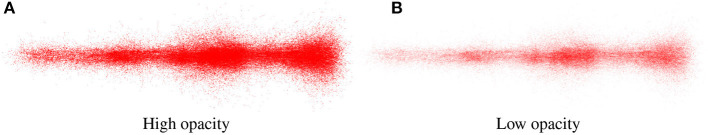
Synapse renderings of the complete data-set showing synapses of a cortical minicolumn with different levels of opacity. **(A)** presents more visual cluttering produced by a high level of opacity. On the other hand, **(B)** shows a lower opacity level. Also, in **(B)**, different clusters with a higher concentration of synapses can be observed along the cortical minicolumn at the left-middle, middle, and right parts of the image.

Figure 6 shows an example of the output obtained when analyzing the spatial positions of synapses. It can be observed that the spatial distribution of these connections shows a specific morphological organization and represent a neuronal superstructure like, in this case, a cortical minicolumn. In addition, by tuning the size and opacity of the particles, the user can reduce the visual clutter generated when dealing with a large number of elements, improving thus the chances for detecting patterns. This can be seen in Figures 6A,B, where the second one facilitates analyzing more populated regions which could not be analyzed that easily in the first one.

This visual aggregation effect, produced by the usage of the particle-rendering approach proposed in this paper, can be also seen in Figure 5. In this scene, the synapses are represented using only the pre-synaptic position encoded in green. It can be noticed that highly populated regions present lighter green colors and are perceived as larger individual clusters. In this figure, it is also shown how SynCoPa allows the analysis of the overall spatial distribution of synapses combining it with the context provided by the neurons' detailed morphologies.

**Figure 7:**
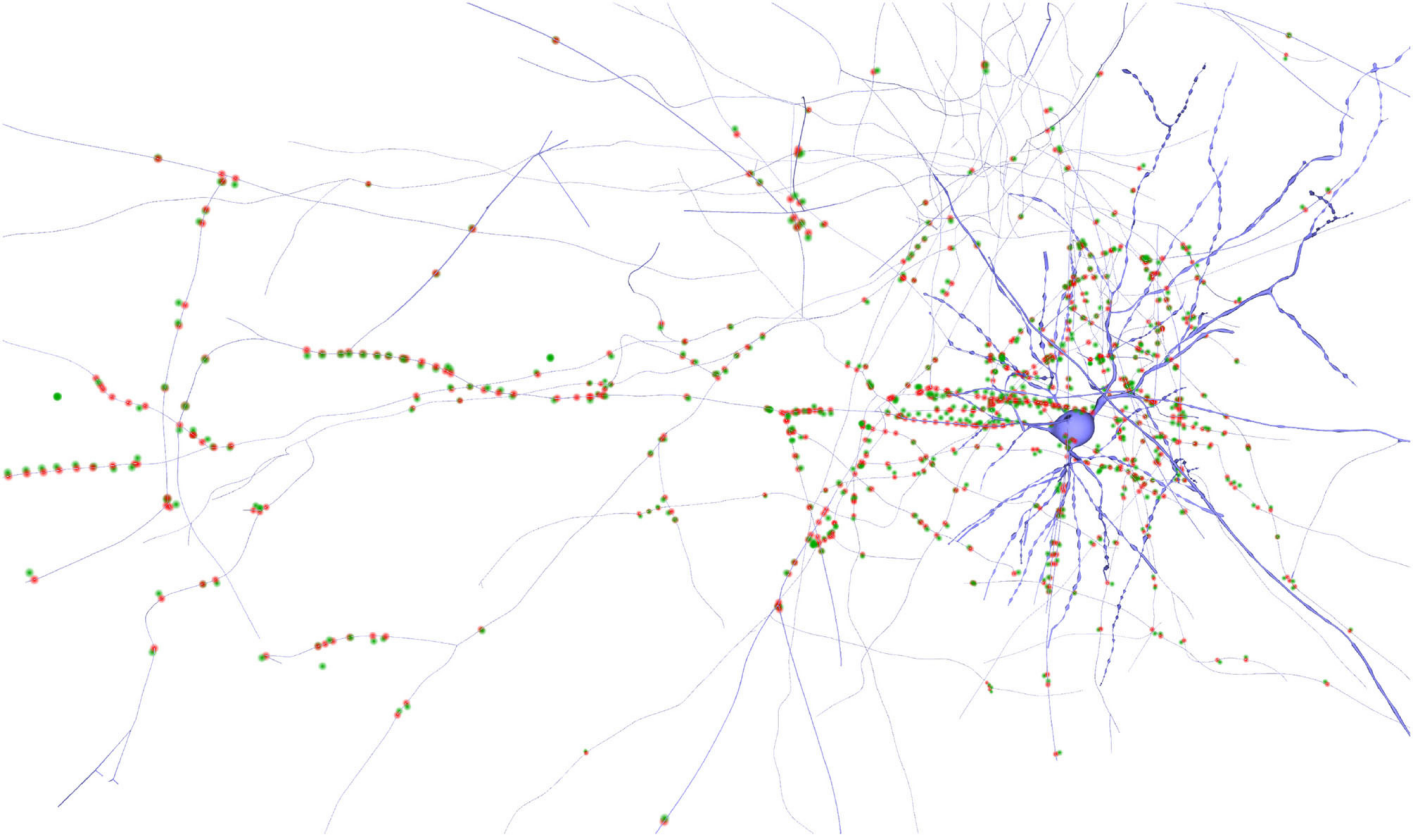
Neuron detailed morphology rendering along with its synapses. The pre-synaptic positions of the synapses are colored in green and the post-synaptic ones in red. Some outliers can be observed as single green points on the left part of the image, too far from the closest dendrite and without a corresponding post-synaptic position in red.

This clustering detection process can also be seen in Figure 7, rendered using two particles per synapse: a green one for the pre-synaptic and a red one for the post-synaptic position. In this case, by analyzing synapse positions together with morphologies it is possible to detect the presence of errors on the positions of some synapses or morphological transformation mismatches. For example, there are some outliers on the left-hand side of the figure, where we can see green particles not having a corresponding post-synaptic red particle close by. Also, it can be seen that some particles seem to be wrongly located, as they are also too far from their closest dendrite. This is an example of how the proposed method can be helpful for detecting possible errors in the data acquisition or dataset composition stages.

### 3.2. Static Path-Highlighting

This use case is based on how connectivity paths can be highlighted to understand the connectivity between neurons. This is of special relevance in cases with high density of branches and synapses, where the user's perception might get overloaded by visual cluttering.

An example of this use case could be as follows: 1) The user loads a circuit and selects the path visualization mode using the selection widget presented in section 2.6. 2) When selecting a pre-synaptic neuron, the application shows its morphology and the whole set of paths leading to the shared synapses of the connecting post-synaptic neurons. 3) The user can tune different visual parameters, colors, opacity, etc., to highlight the paths so they can be seen in a clearer way. Then, the user can select the desired post-synaptic neurons using the selection widget (**Figure 9**). 4) Finally, the application displays the synapses and paths that connect the selected neurons.

**Figure 8:**
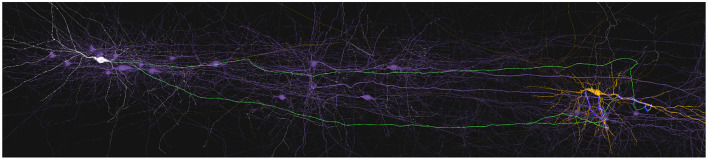
Example of static path-highlighting. In this example, pre-synaptic and post-synaptic neurons have been selected by the user and they are shown in white and orange, respectively. The rest of the neurons in the scene are not selected and are shown in purple. This way, they provide context without interfering in the main features under analysis. The paths between the selected pre-synaptic and post-synaptic neurons are highlighted in green for their pre-synaptic part, axon, and in blue for their post-synaptic part, dendrites. As shown, highlighting paths statically is even effective when the paths between neurons are large enough to require long-distance renders. In these cases, a traditional approach would most likely cause morphological details to be missed due to the visual aliasing effect.

Figure 8 shows a pre-synaptic neuron in white and a post-synaptic neuron in orange. The pre-synaptic part of the path is depicted in green while the post-synaptic part appears in blue. All neurons and neurites not involved in the path are shown in purple. The flexibility of SynCoPa allows the user to change all these colors, as explained in section 2.6. This figure then shows how this static visualization strategy allows to perceive a clear differentiation between the neurites and their sections involved in the connections and the ones which are not. This represents an important advantage when visualizing connectivity on top of realistic morphologies, given the inherent complexity of this kind of data. Connection paths are clearly highlighted from the rest of the scene, even at a long range, where visual aliasing problems interfere directly with the displayed results.

**Figure 9:**
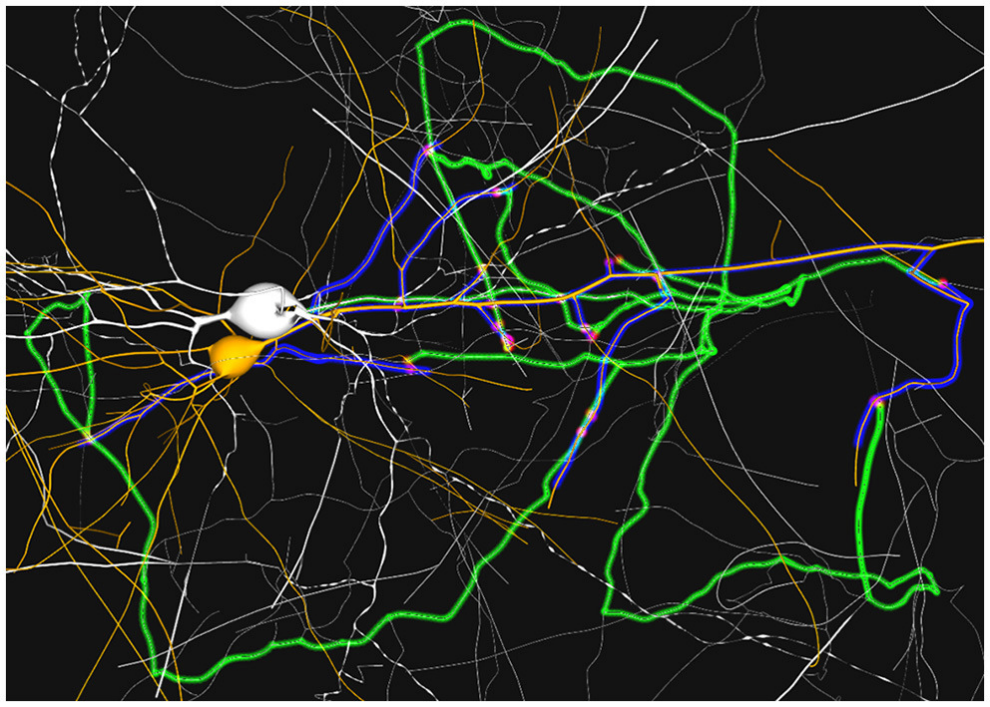
Example of static path-highlighting. The selected pre-synaptic and post-synaptic neurons are shown in white and orange, respectively. Pre-synaptic paths, axon, are highlighted in green and post-synaptic paths, dendrites, in blue. In this detailed view, a closer analysis of paths can be done. Besides, it can be observed that there are post-synaptic parts of paths that overpass the last synapse. These trimming errors are caused by a mismatch between the morphological synaptic distance and the synapse positions.

Moreover, this visualization technique allows the user to interact with the scene, for example by changing the camera viewpoint. It is widely accepted that interaction is always an important point when dealing with complex data. Figure 9 shows how the user can obtain a clear view of the synapse distribution along both the pre and post-synaptic highlighted paths. In this figure, it can also be seen how the pre-synaptic axon, green, is highlighted from the rest of the dendrites of the neuron, presented in white. The unused dendrites from the yellow post-synaptic neuron are clearly differentiated from the highlighted ones in blue.

### 3.3. Color Coding and Filtering of Synapse Attributes

This use case presents some of the analysis procedures that can be carried out when mapping synapse attributes to colors in combination with the execution of interactive filtering operations. This strategy allows users to visually correlate synapse attributes with different spatial properties or patterns, such as synapse location when forming spatial clusters, or their position along the dendrites, branches, and even neural superstructures.

**Figure 10:**
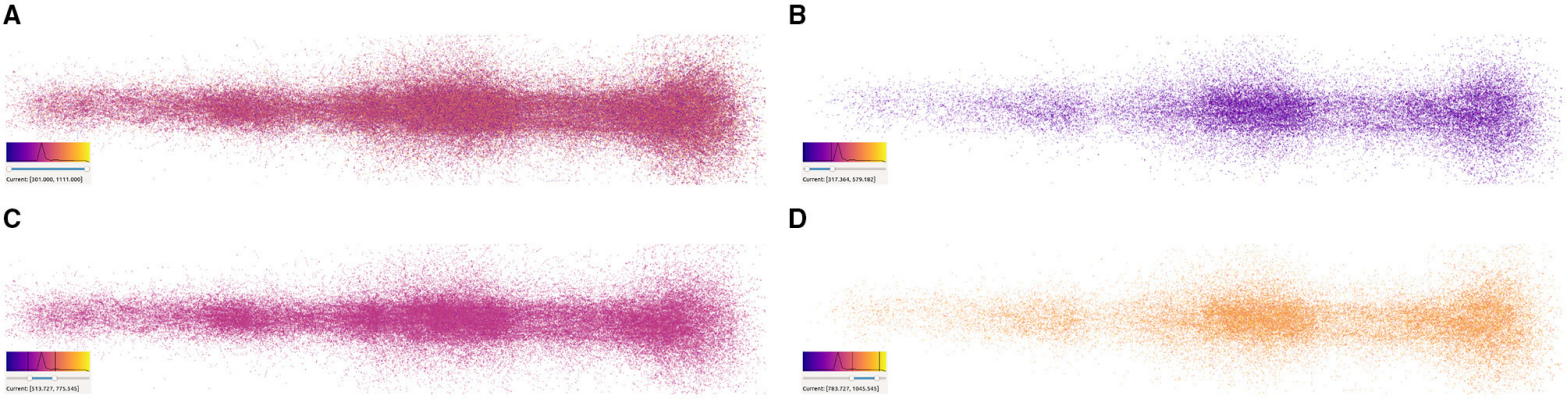
Synapse filtering based on synaptic depression values. **(A)** Shows a full cortical column without filtering, whereas **(B–D)** display the distribution of synapses according to their value and the color composition depending on the filtered range of values. Each figure shows its corresponding color mapping, the histogram, and the selected range on the bottom-left corner.

**Figure 11:**
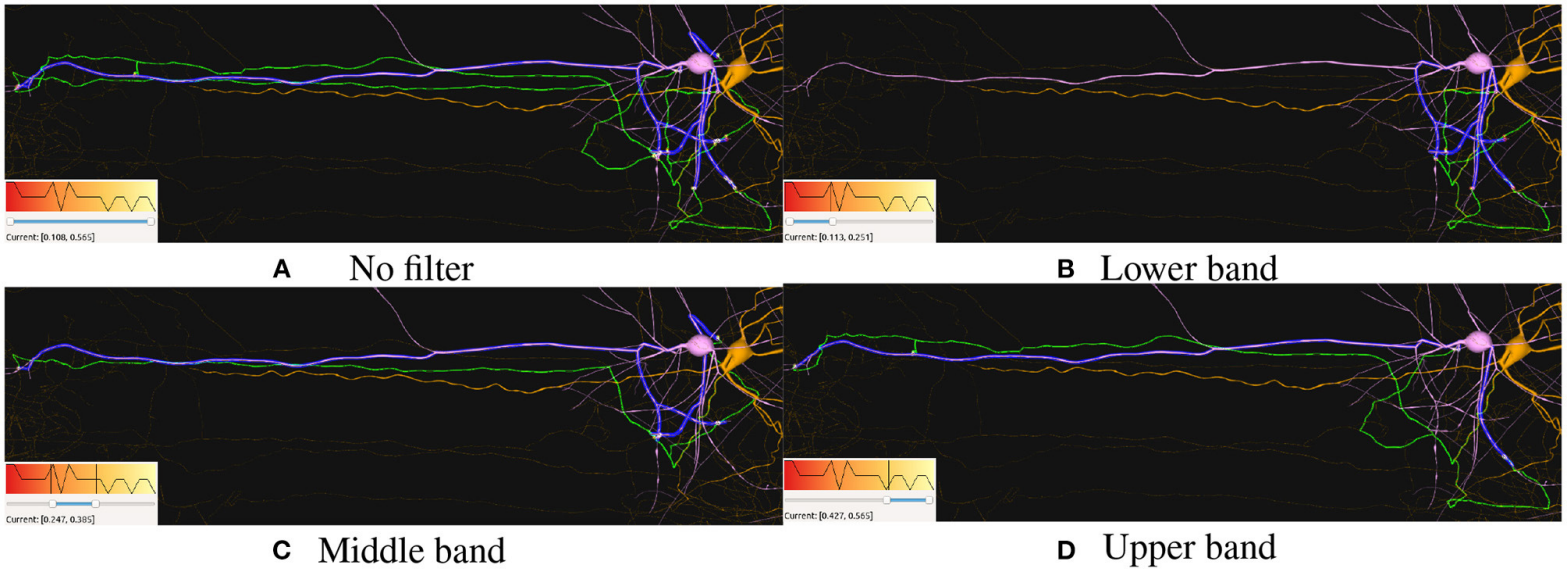
Path filtering based on synaptic conductance values. **(A)** Shows all connecting paths between the pre-synaptic neuron (pink) and the post-synaptic one (orange), highlighting their connecting paths in green and blue, respectively. **(B–D)** Show the different distributions for each range. Each figure shows its corresponding color mapping and the filtering applied at the bottom-left corner.

An example of the workflow associated with this use case could be as follows: 1) The user loads a circuit and the application shows the whole set of synapses; 2) The user selects the synapse mapping option on the widget for controlling their visual appearance and then selects a synapse attribute. This action will make SynCoPa coloring the synapses accordingly as well as displaying the distribution of values as a histogram on top of the gradient widget containing the color palette in use; 3) The user can now change the color palette to a more convenient one according to the distribution of the property values; 4) The user can also activate the filtering option moving the slider to select the desired range of values (Figure 10); 5) With the aim of visually analyzing how the different values are distributed along the neurites, the user can also select a set of neurons so that SynCoPa displays their corresponding morphologies. 6) Finally, the user could also switch to the path highlighting mode, enabling as well the synapse color mapping option. This option can be used for filtering synapses based on the different values of their attributes, allowing users to focus on a specific set of synapses and modify this set interactively. This can be quite useful to the user who, by hiding undesired or non-relevant data, can alleviate visual clutter (Figure 11).

Synapse color-mapping and filtering provide a different way for analyzing the distribution of the values of an attribute along with the morphological organization of the synapses. As it can be observed in Figure 10, the user is able to visually correlate the concentration of values with the spatial distribution of synapses by using a color transfer function. When displaying all the synapses, or a great amount of them, visual clutter can interfere in the users' insight gaining process. This can be seen in Figure 10A, where all synapses are displayed. This is the perfect example to see how the users can interactively filter undesired or nonrelevant information allowing them to visualize ranges independently. In Figure 10B, it can be observed that most synapses are located around their distribution peak, at the middle of the histogram. Figures 10B,D show clusters of values outside the concentration peak which are similar to the ones present in Figure 6B.

Therefore, SynCoPa allows detecting if there are clusters around specific ranges of attribute values. This can be achieved thanks to the visual navigation it provides while combining it with user interaction with the filters features and histograms.

Figure 11 shows how filtering synapses based on their attributes values can also be used for filtering out the resulting paths from each connecting synapse between two or more neurons. Here, it can be seen how the current synapses are color-mapped based on their “Synaptic Conductance” attribute and how paths are filtered when removing the corresponding synapses. This filtering can be used in order to visually correlate different values with the distribution of the connecting elements between neurons. This can be seen in Figure 11B, where no distant connections are present for the lowest Synaptic Conductance values. On the contrary, higher values present in Figures 11C,D show short and long distance connections for the middle and highest values.

### 3.4. Dynamic Path-Highlighting

In this section we present a use case and some examples of the methods proposed in section 2.5.3. In the previous section, it has been shown that visualizing paths using the proposed static visualization technique can help understanding how several neurons connect one another, clearly enhancing the knowledge extraction process. In any case, analyzing the interconnection patterns can still be a difficult task in case where the complexity of the morphologies result in a strong degree of visual clutter. This use case shows how animation can help alleviate perception problems in these types of scenarios.

**Figure 12:**
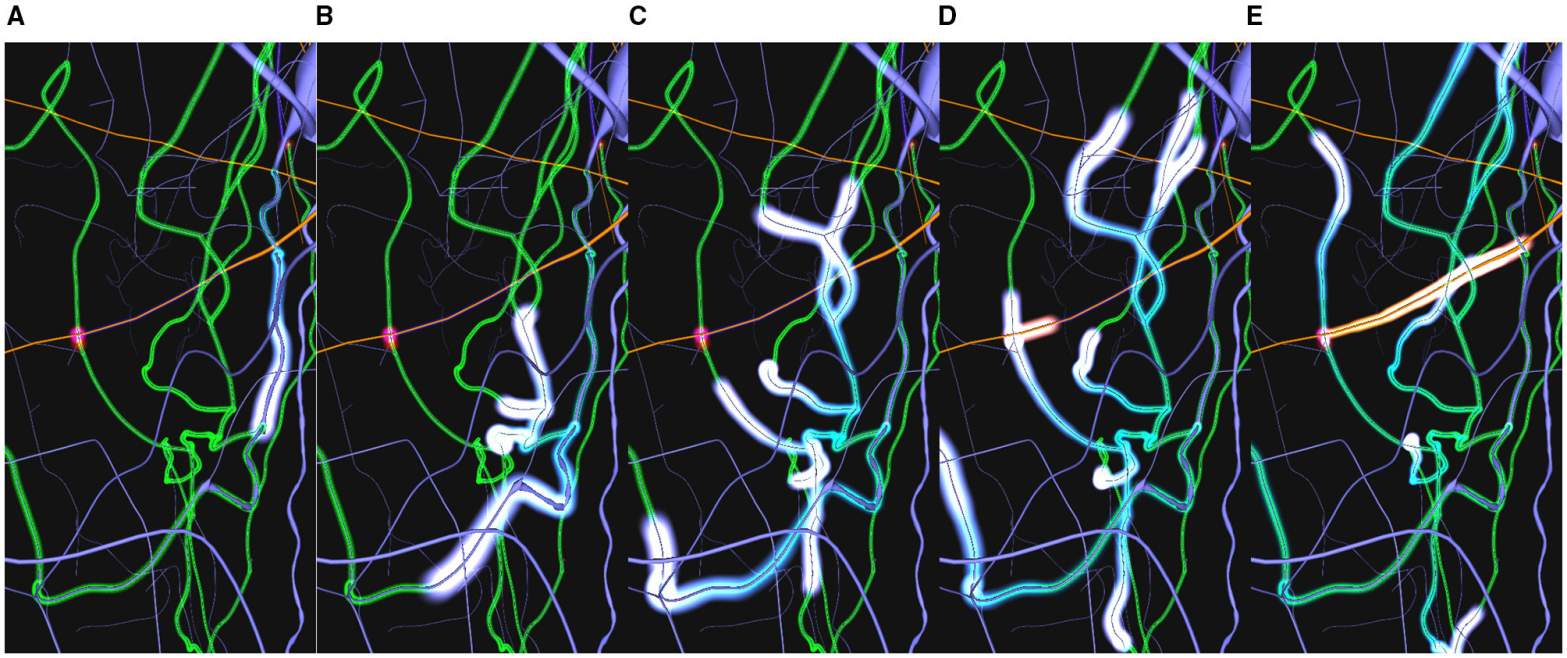
Example sequence of the dynamic highlighting approach. The blue impulse in **(A)** highlights the current path from the blue neuron's soma (top-right). Through both **(B,C)** it can be seen that the initial impulse has split into other impulses on every reached bifurcation. This creates a cascade effect across all available paths (green). Once a synapse has been reached **(D)** a post-synaptic impulse (shown in orange) is created at the synapse position. This impulse follows the dendrite path to the post-synaptic neuron's soma **(E)**.

An example of the workflow associated with this use case could be as follows: 1) The user loads a circuit enabling the path-highlighting mode and then selects the pre-synaptic and post-synaptic neurones. 2) Then, the user can tune different visual parameters and rotate the camera to understand how both neurons are interconnected. 3) By activating the dynamic paths-highlighting, the application displays a motion effect from the pre-synaptic neuron's soma to the post-synaptic's one, branching on every bifurcation and synapse of WS along the neurites' morphology and changing color when reaching a synapse (Figure 12).

The process of highlighting paths dynamically clearly enhances path visualization, even more, when they are complex. One of the clear advantages of using animated sequences is the “pop-out” effect this visual channel generates in the human visual perception system. This motion effect follows the connectivity direction, as it can be seen in Figure 12. As all neurites, and therefore the highlighted paths, tend to interlace (Figure 12A), understanding trajectories and paths gets more difficult with a static highlighting approach. To help alleviate this issue, the moving impulses create an animation that highlights the direction of the paths. This problem is increased when the user is not able to interact using camera navigation. In this case, the trail effect created by the proposed approach also helps understanding the direction of the paths. In Figures 12B,C, it can be observed how impulses split into several impulses for each bifurcations. Figures 12D,E show how a post-synaptic impulse is created when a pre-synaptic impulse reaches a synapse and how the impulses' trails indicate the connectivity directions in static images.

### 3.5. Scope and Limitations

The method effectiveness is determined by several factors, including limitations from hardware and software implementation, data issues or visual constraints.

With respect to morphologies, if complex networks are being considered for analysis, the number of meshes to be rendered simultaneously can compromise the degree of interactivity that can be achieved. Specifically, when running the application with a mono-GPU implementation, as would happen with most desktop or laptop personal computers, the limit might be around 1,000 simultaneous morphologies, but it can really vary depending on their complexity and the amount of detail used (Garcia-Cantero et al., 2017). In this sense, improvements in the NeuroLOTs framework, with the optimization of the method for both single and multiple GPU configurations, might allow an increment of the number of morphologies that can be rendered without compromising interactivity.

Considering synapses and paths, the main limitation comes from the number of particles that have to be rendered. Since the sustained interaction with PReFr (Galindo et al., 2015) on a multi-CPU approach is over 1 million particles, a dataset of over 10,000 simultaneous neurons' synapses could worsen performance. More specifically, static path-highlighting on high complexity scenarios (complex morphologies, long paths or a large number of neurons) will require larger numbers of particles. This can affect performance, and, therefore, significantly compromise interactivity. Nevertheless, the implementation of algorithm on GPUs should increase the number of particles that can be rendered simultaneously to several million. This, along with a multi-GPU configuration, will allow SynCoPa to cope with larger datasets.

In order to have a first approximation to the performance of the prototype presented, we have measured the frames-per second rate (FPS) of the tool under different scene configurations. The computer used was a laptop with 4 cores Intel® Core™ i7-7700HQ CPU (2.80GHz), with 32GB of main memory and a NVIDIA Geforce GTX 1050 GPU. It has to be noticed that SynCoPa is currently a prototype that allows proving the usefulness of the techniques, and has not been optimized yet. Table 1 shows the data collected, where each row presents the number of synapses, the number of neurons, and the FPS achieved when showing the whole set of synapses both with and without the morphologies (labeled as *FPS*_*Syn*+*Mor*_ and *FPS*_*Syn*_, respectively). These configurations are extreme in the sense that the whole dataset is depicted, while in most cases, the analysis tasks presented will typically focus on a subset of the data.

**Table 1:** FPS measurement for different scene configurations.

**Neurons**	**Synapses**	***FPS*_*Syn*_**	***FPS*_*Syn*+*Mor*_**
100	1,849	58	58
200	8,848	47	39
300	18,096	38	27
400	31,752	30	21
500	46,266	24	17
600	71,520	17	14
700	98,388	15	12
800	124,458	11	10
900	143,213	9	9
1,000	182,746	8	8

*The columns “Neuron” and “Synapses” show the size of each configuration. The columns FPS_Syn_ shows the frames-per second (FPS) when rendering the whole set of synapses without morphologies. The FPS numbers when rendering synapses and morphologies are shown in the FPS_Syn+Mor_ column*.

Errors in the data could also compromise the accuracy and effectiveness of the method. Positioning errors could be present in both neurons' morphologies and synapses, when synaptic positions are defined independently. Wrong or poor geometrical transformations could affect neurons' morphological accuracy too. These errors might produce visual artifacts like the ones appearing in Figures 7, 9, as well as other artifacts regarding the trajectory of the paths. Another possible issue is the existence of tracing or positioning errors that generate overlaps between different morphological elements, like neurites and somas. The proposed method can help detect these errors when visualizing the data. However, their correction is not within the scope of this work.

In terms of visualization, there are some limitations regarding alpha blending and the different background configurations. The TAB mode is more effective for visualizing the different color ranges when mapping synapses' attributes to colors for both light and dark backgrounds. On the other hand, the AAB reduces significantly its effectiveness with light backgrounds since the accumulation of color saturates to white. As the concentration of elements is key for computing the resulting color, using AAB challenges the accuracy of the colors or elements concentration. As a consequence, the path-highlighting method in both its static and dynamic variations proves to be less effective over light backgrounds. On the contrary, paths highlighting in both static and dynamic approaches offer better effectiveness with both AAB and dark backgrounds. AAB mode results are very effective for color differentiation in tasks, such as observing synapses concentration due to the use of light colors over dark backgrounds where the saturation also indicates concentration, enhancing the overall understanding of the scene.

## 4. Conclusions and Future Work

This article presents SynCoPa, a tool that allows users to interactively explore and analyze detailed morphological and structural information of neuronal circuits at the connectome level (fulfilling FR1). More specifically, the work presented here focuses on two analysis tasks: 1) Interactively analyzing the relationships between the detailed morphologies of a neuronal circuit and its synapses spatial location and attributes; 2) Studying connecting paths between neurons and the relationship of these paths with morphological features.

The proposed synapse representation using particle-based rendering has turned out to be very effective. This representation can be tuned using different visual properties to enhance the users' perception. For example, alpha blending can be adjusted to help understanding the spatial distribution of synapses. This is specially useful in cases with a large amount of elements being displayed at the same time, such as several hundreds of thousands of synapses being shown simultaneously. Moreover, the adjustment of visual parameters clearly enhances the perception of patterns, concentrations of elements, and even outliers and positioning errors (contributing to the achievement of functional requirements FR2, FR3).

Furthermore, the proposed method allows mapping synapse attributes to colors. In addition, and in combination with interactive user-defined filtering, it also allows to effectively understand the distribution of values in a specific region and at different levels of detail, for example at neuron level or at population level (fulfilling functional requirement FR3).

Highlighting paths statically provide an effective perception of neuron connectivity, especially when visualizing a large amount of neurites, or when their morphologies are very complex. In these cases, visual clutter muddles the users' ability to analyze the data as discussed previously in this article. The proposed method is also effective for highlighting paths at long camera distances, where the visual aliasing effect is more remarkable. On the other hand, highlighting paths dynamically creates animations that effectively convey the connectivity direction and enhance the understanding of the scene even without any type of camera interaction. We have concluded that path highlighting (either static or dynamic) provides a pop-out effect of tracing or positioning errors when trajectories do not match paths or synapses' positions (fulfilling FR4).

With respect to the limitations found in SynCoPa, we can conclude that the main ones could be alleviated by improving the computing performance of the underlying rendering technologies supporting SynCoPa.

In summary, this article proposes several novel methods for exploring and analyzing synapse distributions, together with their attributes and the connectivity paths between neurons. These methods can help neuroscientists to gain insight into complex scenarios combining both connectivity and detailed morphologies. In addition, they can also help with the detection of tracing and positioning errors.

Regarding the interaction with the applications, at this moment we only provide a filtering option based on mapping certain attributes to different colors. However, we are already working on the development of a few more filtering options to reduce the amount of neurons, synapses, and paths shown according to different properties, so that users will be able to select certain elements, for example using drag and drop operations, from the dataset to focus on a more specific WS.

It would also be very interesting to allow SynCoPa to perform an automatic size adjustment for different sections of the neurites. This would help make sense of the images presented to the user, since different neurite sections along interconnection paths have different sizes. However, the particles deployed are always presented with the same size.

Expanding the proposed method to perform quantitative analysis tasks could also be very interesting. This could provide several instruments for a detailed numeric analysis of the morphologies and connectivity elements, as well as better validation mechanisms of the data. These quantitative analysis tools could be useful to improve the data extraction and generation processes in previous steps of the brain study.

Finally, the methods proposed could be improved making it possible to load a broader range of datasets, types, and formats to show and analyze their morphological details together with the available neuronal connections. Also, CPU and GPU optimizations would be beneficial in order to cope with larger datasets.

## Data Availability Statement

The original contributions presented in the study are included in the article/Supplementary Material, further inquiries can be directed to the corresponding author/s.

## Author Contributions

SG, PT, and OR worked on the design of the system. SG and PT worked on the implementation. All authors conceived of the project, reviewed, contributed, and approved the final version of the manuscript.

## Funding

The research leading to these results has received funding from the Spanish Ministry of Economy and Competitiveness under grants C080020-09 (Cajal Blue Brain Project, Spanish partner of the Blue Brain Project initiative from EPFL) and TIN2017-83132, and the Spanish Ministry of Science and Innovation under grants PID2020-113013RB-C21 and PID2020-113013RB-C22, as well as from the European Union's Horizon 2020 Framework Programme for Research and Innovation under the Specific Grant Agreements No. 785907 (Human Brain Project SGA2) and 945539 (Human Brain Project SGA3).

## Conflict of Interest

The authors declare that the research was conducted in the absence of any commercial or financial relationships that could be construed as a potential conflict of interest.

## Publisher's Note

All claims expressed in this article are solely those of the authors and do not necessarily represent those of their affiliated organizations, or those of the publisher, the editors and the reviewers. Any product that may be evaluated in this article, or claim that may be made by its manufacturer, is not guaranteed or endorsed by the publisher.

## Supplementary Material

Four demo videos are provided for this paper:

- Use case 1 (Available online at: https://youtu.be/pZ7WVpW8G-4).
- Use case 2 (Available online at: https://youtu.be/XucXTeB0jMI).
- Use case 3 (Available online at: https://youtu.be/AcCR5Mxgi_k).
- Use case 4 (Available online at: https://youtu.be/aGD_hbXnB7k).

## References

[B1] Akar N. A. Cumming B. Karakasis V. Küsters A. Klijn W. Peyser A.. (2019). Arbor - a morphologically-detailed neural network simulation library for contemporary high-performance computing architectures, in 2019 27th Euromicro International Conference on Parallel, Distributed and Network-Based Processing (PDP) (Pavia), 274–282.

[B2] Al-Awami A. K. Beyer J. Strobelt H. Kasthuri N. Lichtman J. W. Pfister H.. (2014). Neurolines: a subway map metaphor for visualizing nanoscale neuronal connectivity. IEEE Trans. Vis. Comput. Graph. 20, 2369–2378. 10.1109/TVCG.2014.2346312 26356951

[B3] Bartram L. (1997). Can motion increase user interface bandwidth in complex systems?, in Proceedings of the IEEE International Conference on Systems, Man and Cybernetics (Orlando, FL).

[B4] Beyer J. Al-Awami A. Kasthuri N. Lichtman J. Pfister H. Hadwiger M. (2013). Connectomeexplorer: query-guided visual analysis of large volumetric neuroscience data. IEEE Trans. Vis. Comput. Graph. 19, 2868–2877. 10.1109/TVCG.2013.142 24051854 PMC4296725

[B5] Böttger J. Schäfer A. Lohmann G. Villringer A. Margulies D. S. (2014). Three-dimensional mean-shift edge bundling for the visualization of functional connectivity in the brain. IEEE Trans. Vis. Comput. Graph. 20, 471–480. 10.1109/TVCG.2013.114 23959625

[B6] Bruckner S. Soltészová V. Gröller M. E. Hladůvka J. Bühler K. Yu J.. (2009). Braingazer - visual queries for neurobiology research. IEEE Trans. Vis. Comput. Graph. 15, 1497–1504. 10.1109/TVCG.2009.121 19834226

[B7] Cannon R. Turner D. Pyapali G. Wheal H. (1998). An on-line archive of reconstructed hippocampal neurons. J. Neurosci. Methods 84, 49–54. 9821633 10.1016/s0165-0270(98)00091-0

[B8] Carnevale T. Hines M. (2006). The NEURON Book. Cambridge, UK: Cambridge University Press.

[B9] Collins F. Prabhakar A. (2013). BRAIN Initiative Challenges Researchers to Unlock Mysteries of Human Mind. Available online at: http://www.whitehouse.gov/blog/2013/04/02/brain-initiative-challenges-researchers-unlock-mysteries-human-mind.

[B10] Combrisson E. Vallat R. O'Reilly C. Jas M. Pascarella A. Saive A.. (2019). Visbrain: a multi-purpose gpu-accelerated open-source suite for multimodal brain data visualization. Front. Neuroinform. 13:14. 10.3389/fninf.2019.00014 30967769 PMC6439346

[B11] Conte G. Ye A. Almryde K. Ajilore O. Leow A. Forbes A. G. (2016). Intrinsic geometry visualization for the interactive analysis of brain connectivity patterns, in Visualization and Data Analysis (VDA) Proceedings of the SI&T International Symposium on electronic Imaging, Science and Technology (San Francisco, CA: Society for Imaging and Technology), 1–8.

[B12] Euán C. Sun Y. Ombao H. (2019). Coherence-based time series clustering for statistical inference and visualization of brain connectivity. Ann. Appl. Stat. 13, 990–1015. 10.1214/18-AOAS122518320210

[B13] Evanko D. Pastrana E. (2013). Why mapping the brain matters. Nat. Methods 10:447. 10.1038/nmeth.2513 23866326

[B14] Friston K. J. (1994). Functional and effective connectivity in neuroimaging: a synthesis. Hum. Brain Mapp. 2, 56–78.

[B15] Friston K. J. (2011). Functional and effective connectivity: a review. Brain Connect. 1, 13–36. 10.1089/brain.2011.0008 22432952

[B16] Fujiwara T. Chou J. McCullough A. M. Ranganath C. Ma K. (2017). A visual analytics system for brain functional connectivity comparison across individuals, groups, and time points, in 2017 IEEE Pacific Visualization Symposium (PacificVis) (Seoul), 250–259.

[B17] Galindo S. E. Toharia P. Lopez-Moreno J. Robles O. D. Pastor L. (2015). PREFR: a flexible particle rendering framework, in XXV Spanish Computer Graphics Conference, CEIG 2015, Benicàssim (Castellón), Spain, July 1–3, 2015, eds Sbert M. Lopez-Moreno J. (Eurographics Association), 9–17.

[B18] Galindo S. E. Toharia P. Robles O. D. Pastor L. (2016). ViSimpl: multi-view visual analysis of brain simulation data. Front. Neuroinform. 10:44. 10.3389/fninf.2016.00044 27774062 PMC5054003

[B19] Galindo S. E. Toharia P. Robles O. D. Ros E. Pastor L. Garrido J. A. (2020). Simulation, visualization and analysis tools for pattern recognition assessment with spiking neuronal networks. Neurocomputing 400, 309–321. 10.1016/j.neucom.2020.02.114

[B20] Garcia-Cantero J. J. Brito J. P. Mata S. Bayona S. Pastor L. (2017). Neurotessmesh: a tool for the generation and visualization of neuron meshes and adaptive on-the-fly refinement. Front. Neuroinform. 11:38. 10.3389/fninf.2017.00038 28690511 PMC5479896

[B21] Hastings E. J. Guha R. K. Stanley K. O. (2009). Interactive evolution of particle systems for computer graphics and animation. IEEE Trans. Evol. Comput. 13, 418–432. 10.1109/TEVC.2008.200426127295638

[B22] He B. Dai Y. Astolfi L. Babiloni F. Yuan H. Yang L. (2011). Econnectome: a matlab toolbox for mapping and imaging of brain functional connectivity. J. Neurosci. Methods 195, 261–269. 10.1016/j.jneumeth.2010.11.015 21130115 PMC3244474

[B23] Hernando J. B. Biddiscombe J. Bohara B. Eilemann S. Schürmann F. (2013). Practical parallel rendering of detailed neuron simulations, in Eurographics Symposium on Parallel Graphics and Visualization, eds Marton F. Moreland K. (Girona: The Eurographics Association).

[B24] Koffka K. (2014). Principles Of Gestalt Psychology. Abingdon: Mimesis International.

[B25] Lin C.-Y. Tsai K.-L. Wang S.-C. Hsieh C.-H. Chiang A.-S. (2011). The neuron navigator: exploring the information pathway through the neural maze, in PacificVis, eds Battista G. D. Fekete J. D. Qu H. (Hong Kong: IEEE), 35–42.

[B26] LoCastro E. Kuceyeski A. Raj A. (2014). Brainography: an atlas-independent surface and network rendering tool for neural connectivity visualization. Neuroinformatics 12, 355–359. 10.1007/s12021-013-9206-1 24081830

[B27] Lorach T. (2007). Soft Particles. Technical Report, NVIDIA White Paper, Santa Clara, CA.

[B28] Ma K.-L. Muelder C. W. (2013). Large-scale graph visualization and analytics. Computer 46, 39–46. 10.1109/MC.2013.24227295638

[B29] Markram H. (2006). The blue brain project. Nat. Rev. Neurosci. 7, 153–160. 10.1038/nrn1848 16429124

[B30] Markram H. Muller E. Ramaswamy S. Reimann M. Abdellah M. Sanchez C.. (2015). Reconstruction and simulation of neocortical microcircuitry. Cell 163, 456–492. 10.1016/j.cell.2015.09.029 26451489

[B31] Mijalkov M. Kakaei E. Pereira J. B. Westman E. Volpe G. (2017). Braph: a graph theory software for the analysis of brain connectivity. bioRxiv. 12, 1–23. 10.1371/journal.pone.0178798 28763447 PMC5538719

[B32] Morgan J. L. Lichtman J. W. (2013). Why not connectomics? Nat. Methods 10, 494–500. 10.1038/nmeth.2480 23722208 PMC4184185

[B33] Mullen T. Kothe C. Chi Y. M. Ojeda A. Kerth T. Makeig S.. (2013). Real-time modeling and 3d visualization of source dynamics and connectivity using wearable EEG, in 2013 35th Annual International Conference of the IEEE Engineering in Medicine and Biology Society (EMBC) (Osaka), 2184–2187. 10.1109/EMBC.2013.6609968 PMC4119601 24110155

[B34] Nordlie E. Plesser H. E. (2010). Visualizing neuronal network connectivity with connectivity pattern tables. Front. Neuroinform. 3:39. 10.3389/neuro.11.039.2009 20140265 PMC2816167

[B35] Peyser A. Sinha A. Vennemo SB. Ippen T. Jordan J. Graber S.. (2017). Nest 2.14.0.

[B36] Rubinov M. Sporns O. (2010). Complex network measures of brain connectivity: uses and interpretations. Neuroimage 52, 1059–1069. 10.1016/j.neuroimage.2009.10.003 19819337

[B37] Senk J. Carde C. Hagen E. Kuhlen T. W. Diesmann M. Weyers B. (2018). Viola'a multi-purpose and web-based visualization tool for neuronal-network simulation output. Front. Neuroinform. 12:75. 10.3389/fninf.2018.00075 30467469 PMC6236002

[B38] Shneiderman B. (1996). The eyes have it: a task by data type taxonomy for information visualizations, in Proceedings 1996 IEEE Symposium on Visual Languages (Boulder, CO), 336–343.

[B39] Sporns O. Tononi G. Kötter R. (2005). The human connectome: a structural description of the human brain. PLoS Comput. Biol. 1:e42. 10.1371/journal.pcbi.0010042 16201007 PMC1239902

[B40] van Dixhoorn A. F. Milles J. van Lew B. Botha C. P. (2012). Braincove: a tool for voxel-wise fmri brain connectivity visualization, in Proceedings of the Eurographics Workshop on Visual Computing for Biology and Medicine, eds Ropinski T. Ynnerman A. Botha C. Roerdink J. (Norrköping: The Eurographics Association), 99–106.

[B41] van Dixhoorn A. F. Vissers B. H. Ferrarini L. Milles J. Botha C. P. (2010). Visual analysis of integrated resting state functional brain connectivity and anatomy, in Proceedings of the 2nd Eurographics Conference on Visual Computing for Biology and Medicine (Leipzig: The Eurographics Association), 57–64.

